# Nanotechnology for fungal pathogen control in crops: innovations, public health impacts, and disease prevention

**DOI:** 10.3389/ffunb.2025.1653214

**Published:** 2025-09-01

**Authors:** Matthew Chidozie Ogwu, Sylvester Chibueze Izah

**Affiliations:** ^1^ Goodnight Family Department of Sustainable Development, Appalachian State University, Boone, NC, United States; ^2^ Department of Community Medicine, Faculty of Clinical Sciences, Bayelsa Medical University, Yenagoa, Bayelsa State, Nigeria

**Keywords:** fungal pathogens, sustainable agriculture, crop protection, nanopesticides, food security, antimicrobial resistance, public health

## Abstract

Fungal pathogens continue to devastate global agriculture, causing significant crop losses, compromising food security, and posing emerging threats to public health. This paper critically examines the revolutionary role of nanotechnology-driven innovations in combating fungal diseases in crops, offering an integrative framework that bridges plant health, environmental sustainability, and human well-being. We synthesize recent advancements in agricultural nanomaterials, including silver, zinc oxide, and copper oxide nanoparticles, as well as green-synthesized nanoformulations. We examine their antifungal mechanisms, including membrane disruption, induction of oxidative stress, targeted delivery, and inhibition of spore germination. The review highlights how nanosensors can facilitate early detection of pathogens, while nano-enabled packaging and innovative delivery systems prevent post-harvest contamination and extend shelf life. Crucially, we underscore the public health benefits of reduced chemical pesticide use, lowered mycotoxin exposure, and the potential for mitigating antimicrobial resistance. The paper advances the discourse on environmentally responsible, high-precision disease control strategies in agriculture by linking nanotechnology to broader sustainability goals. Furthermore, we identify key challenges, including regulatory ambiguity, ecotoxicological concerns, and barriers to equitable adoption, especially among smallholder farmers in the Global South. This paper contributes a forward-looking agenda for integrating nanotechnology into holistic pest management systems through inclusive policies, interdisciplinary research, and stakeholder-driven implementation pathways. Overall, this review positions nanotechnology as a transformative tool in reengineering crop protection paradigms that align innovation with sustainability, resilience, and public health imperatives in the face of escalating global challenges.

## 1 Introduction

The advent of nanotechnology represents a revolutionary shift in agricultural practices, particularly in managing fungal pathogens that threaten crop yield and quality. Nanotechnology may be defined as manipulating matter on an atomic, molecular, and supramolecular scale to offer promising innovations for sustainable agriculture (Ray et al., 2023). The applications of nanotechnology in agriculture encompass enhanced disease management, precision farming, and advanced delivery systems for agrochemicals, which are essential components for improving crop resilience to pathogens (Kumar et al., 2023; Shahid et al., 2023). As the global agricultural landscape increasingly confronts challenges posed by climate change and pest resistance, the role of nanotechnology in fostering sustainable farming practices gains significance (Das et al., 2021; Vijayreddy et al., 2023).

Emerging threats from plant fungal pathogens have escalated concerns regarding food security, given their capacity to decimate crops and reduce overall agricultural productivity (Bebber and Gurr, 2015; Kang et al., 2021). The estimated annual losses due to fungal diseases can reach approximately 20% of agricultural yield, impacting global food supply chains and increasing food prices (Godfray et al., 2016; Almeida et al., 2019; Ogwu et al., 2024). Additionally, the rising incidence of pathogen resistance to conventional fungicides exacerbates the challenges faced by farmers, necessitating innovative solutions that nanotechnology can provide (Azevedo et al., 2015; Ogwu and Izah, 2023). These solutions could redefine conventional disease management approaches by integrating bioactive nanomaterials that can disrupt fungal growth mechanisms (Ogwu and Osawaru, 2022; Shahid et al., 2023).

Fungal diseases significantly hinder agriculture, prompting economists, agronomists, and public health officials to assess their broader implications beyond crop losses (Suryani et al., 2023). The global impact of plant fungal pathogens, as evidenced by the proliferation of diseases such as rust, blight, and mildew, underlines the urgency for effective management strategies (Bebber and Gurr, 2015). Pathogens like *Aspergillus fumigatus* threaten crops and have also been implicated in the emergence of antifungal resistance within human pathogens, creating a nexus between agricultural practices and public health (Kang et al., 2021). The occurrence of pathogenic strains resistant to azole fungicides poses significant challenges for both crop management and clinical treatment (Azevedo et al., 2015; Shen et al., 2022). The conventional reliance on synthetic fungicides has been insufficient, primarily due to fungicide resistance and the detrimental impact of chemical residues on soil health and microbial diversity (Dutta et al., 2023; Hamid and Saleem, 2022). Researchers highlight that nitrogen and phosphorus fertilization practices have unintentionally favored pathogenic fungi, illustrating the complex interactions within agricultural ecosystems that can lead to increased disease severity (Lekberg et al., 2021). Therefore, advancements in nanotechnology that target these specific pathways promise more effective and sustainable disease control methods, which can be integrated into current agricultural frameworks (Vijayreddy et al., 2023; Ogwu et al., 2024; 2025).

The interrelationship between plant health, crop diseases, and public health is multifaceted. Effective management of crop diseases is vital for food security and has broader implications for environmental and human health (Bratovčić et al., 2023). The circulatory impact of agricultural practices on public health is evident in the context of zoonotic diseases and the frequent human exposure to harmful pesticides, commonly used in traditional farming methods. The transition towards nanotechnology applications in agriculture could mitigate these risks by offering targeted delivery systems that minimize chemical usage while maximizing pest and pathogen control (Sharma et al., 2024). The environmental repercussions of conventional agriculture also heighten public health concerns. Pesticide residues frequently contaminate soil and water, posing significant threats to both terrestrial and aquatic ecosystems (Dutta et al., 2023; Hamid and Saleem, 2022). Agriculture can utilize nanotechnology to enhance the efficacy of biocontrol agents and reduce chemical inputs by leveraging the unique properties of nanomaterials, such as their high surface-area-to-volume ratio (Shahid et al., 2023). This change helps maintain ecosystem health, which is essential for long-term agricultural output and, in turn, for public health outcomes, as well as for disease control. Therefore, embracing nanotechnology can reduce reliance on harmful synthetic chemicals, aligning agricultural practices with the Sustainable Development Goals (Ogwu et al. 2024). Nanotechnology has the potential to enhance food safety by reducing post-harvest losses and increasing disease resistance, leading to higher-quality produce and improved public health outcomes (Bratovčić et al., 2023).

This review offers a critical and integrative examination of the emerging role of nanotechnology in controlling fungal pathogens that affect crop systems, with a particular focus on its implications for sustainable agriculture, food safety, and public health. Unlike existing reviews that predominantly address the synthesis and application of nanomaterials in crop protection (Chhipa, 2017; Worrall et al., 2018), our work offers a transdisciplinary perspective that situates nanotechnological innovations within a broader context of global health and environmental sustainability. It uniquely emphasizes the dual relevance of fungal disease management and public health, especially in low- and middle-income countries where the burden of foodborne mycotoxins and antimicrobial resistance remains high. The review synthesizes cutting-edge developments in the design and deployment of nanomaterials, including metallic nanoparticles, nanoemulsions, and stimuli-responsive delivery systems for effective and targeted fungal control. It elucidates their underlying antifungal mechanisms, such as cell membrane disruption, oxidative stress induction, and enzymatic inhibition, while also evaluating their capacity to overcome conventional resistance pathways. Furthermore, we explore nanotechnology-enabled strategies for early pathogen detection, post-harvest disease mitigation, and intelligent food packaging that collectively enhance food quality and shelf life. Crucially, the review addresses key issues related to equity, ecology, and regulations that are crucial to the responsible application of nanotechnologies. This review provides a thorough synthesis that connects nanoscience, plant pathology, and public health, advancing a novel framework for the inclusive and sustainable integration of nanotechnology into international crop protection efforts.

## 2 Nanotechnology approaches for managing fungal pathogens

**Figure 1:**
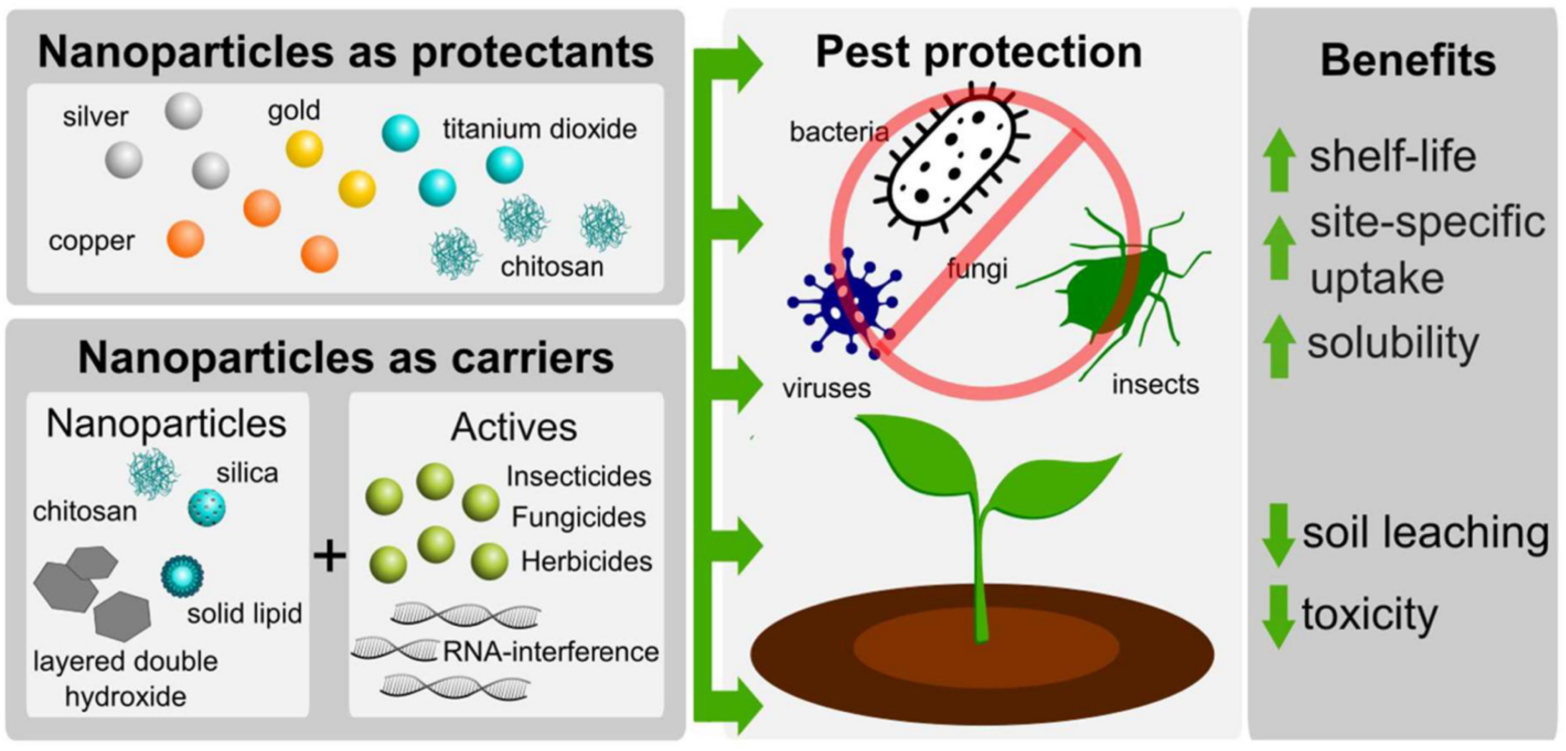
Nanoparticles as smart tools for crop pest protection and agrochemical delivery. Source: Worrall et al. (2018), reproduced under CC BY 4.0 license.

The burgeoning field of nanotechnology has been increasingly recognized for its role in managing fungal pathogens, particularly through the use of various nanomaterials (**Figure 1**). **Figure 1** illustrates the dual role of nanoparticles in agriculture as both protectants and carriers. As protectants, nanoparticles such as silver, copper, gold, titanium dioxide, and chitosan directly inhibit plant pathogens, including bacteria, viruses, fungi, and insect pests. As carriers, various nanomaterials, including chitosan, silica, solid lipid nanoparticles, and layered double hydroxides, enhance the delivery and efficacy of active agents such as insecticides, fungicides, herbicides, and RNA-interference molecules (Worrall et al., 2018). Nanodelivery systems offer multiple agronomic benefits, including increased shelf life, targeted delivery, enhanced solubility, and reduced toxicity and environmental leaching, thereby contributing to sustainable and precise pest management strategies in modern agriculture (**Figure 1**; Worrall et al., 2018).

These materials differ in composition, structure, and functional mechanisms, offering a wide range of benefits from enhanced efficacy to environmental sustainability. **Table 1** categorizes key types of antifungal nanomaterials, highlighting their representative examples, modes of action, application methods, advantages, and corresponding references. This classification provides a foundational understanding of how nanotechnology can be strategically leveraged to combat fungal pathogens in crops while reducing chemical inputs, improving delivery precision, and supporting sustainable disease management practices. Among these materials, nanoparticles such as silver, zinc oxide, and copper oxide have been shown to exhibit antifungal properties.

**Table 1:** Nanotechnology approaches for managing fungal pathogens in agriculture.

Nanomaterial type	Examples	Mechanism of action	Application mode	Advantages	References
Metallic Nanoparticles	Silver (Ag), Zinc Oxide (ZnO), Copper Oxide (CuO)	Disrupt cell walls/membranes, induce ROS (reactive oxygen species), and inhibit enzymatic activity	Foliar sprays, seed coatings, soil amendments	Broad-spectrum antifungal activity, low resistance development	Girma (2023)
Nanocarriers for Fungicides	Lipid-based NPs, polymeric NPs, silica NPs	Controlled release of active ingredients, targeted delivery to infection sites	Encapsulated fungicide sprays, root zone delivery	Reduced dosage, sustained release, minimized non-target effects	Kutawa et al. (2021); Kumar et al. (2022)
Nanostructured Materials	Carbon nanotubes (CNTs), nanofibers, and quantum dots	Physical interaction with fungal spores, blockage of nutrient uptake	Coatings on surfaces, plant-based carriers	Mechanical inhibition, enhanced stability	Patel et al. (2020); Safdar et al. (2022)
Green Synthesized Nanoparticles	Plant-mediated AgNPs, fungal/myco-nanoparticles	Bio-compatible antifungal action via phytochemicals and metallic ions	Eco-friendly foliar and soil application	Environmentally sustainable, reduced toxicity	Borehalli Mayegowda et al. (2023); Singh et al. (2023)
Nanoemulsions	Essential oils + surfactant nanoemulsions	Membrane disruption, inhibition of spore germination	Spray or dip application	Natural, biodegradable, and synergistic with other biocontrol agents	Chang et al. (2022); Mosa et al. (2023)
Magnetic Nanoparticles	Fe₃O₄ NPs, iron oxide NPs	Magnetic targeting, potential for pathogen trapping	Combined with magnetically guided delivery systems	Precise targeting, potential for recycling	Shen et al. (2018)

**Figure 2:**
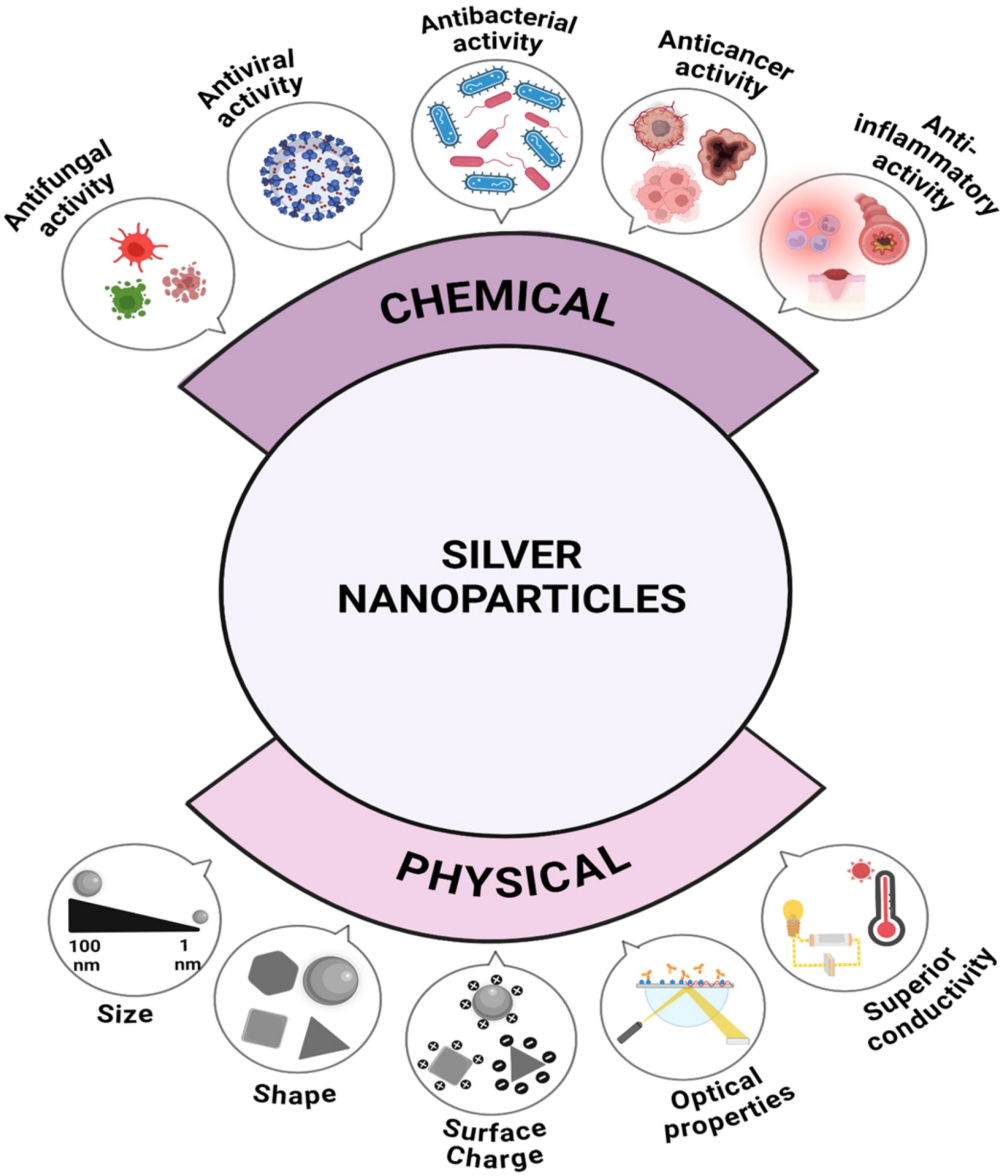
Multifunctional properties of silver nanoparticles: chemical and physical perspectives. Source: Duman et al. (2024), reproduced under CC BY 4.0 license.

Silver nanoparticles (AgNPs) are among the most extensively studied nanomaterials due to their broad-spectrum biological activities and unique physicochemical characteristics (**Figure 2**). Chemically, AgNPs exhibit potent antifungal, antiviral, antibacterial, anticancer, and anti-inflammatory activities, making them valuable in medicine, agriculture, and environmental management. Physically, nanoscale size, surface charge, shape variability, optical behavior, and superior conductivity contribute to their versatility and effectiveness (**Figure 2**). These traits make AgNPs promising candidates for next-generation solutions across disciplines, including plant pathology, healthcare, and nanotechnology-enabled diagnostics. AgNPs are particularly noted for their broad-spectrum antimicrobial activities, including efficacy against diverse fungal strains such as *Candida* and *Aspergillus* species (Santos et al., 2021; Wahab et al., 2023). Research reveals that these nanoparticles can disrupt the cell membranes of fungi, which is pivotal in inhibiting growth and inducing cell death via apoptosis (Pachaiappan et al., 2021; Mohsen et al., 2022). This phenomenon is primarily attributed to their ability to release silver ions, which interact with cellular components, leading to oxidative stress and functional impairment of pathogenic cells (Mohammed et al., 2018; Ikhajiagbe et al., 2020; Wahab et al., 2023).

**Figure 3:**
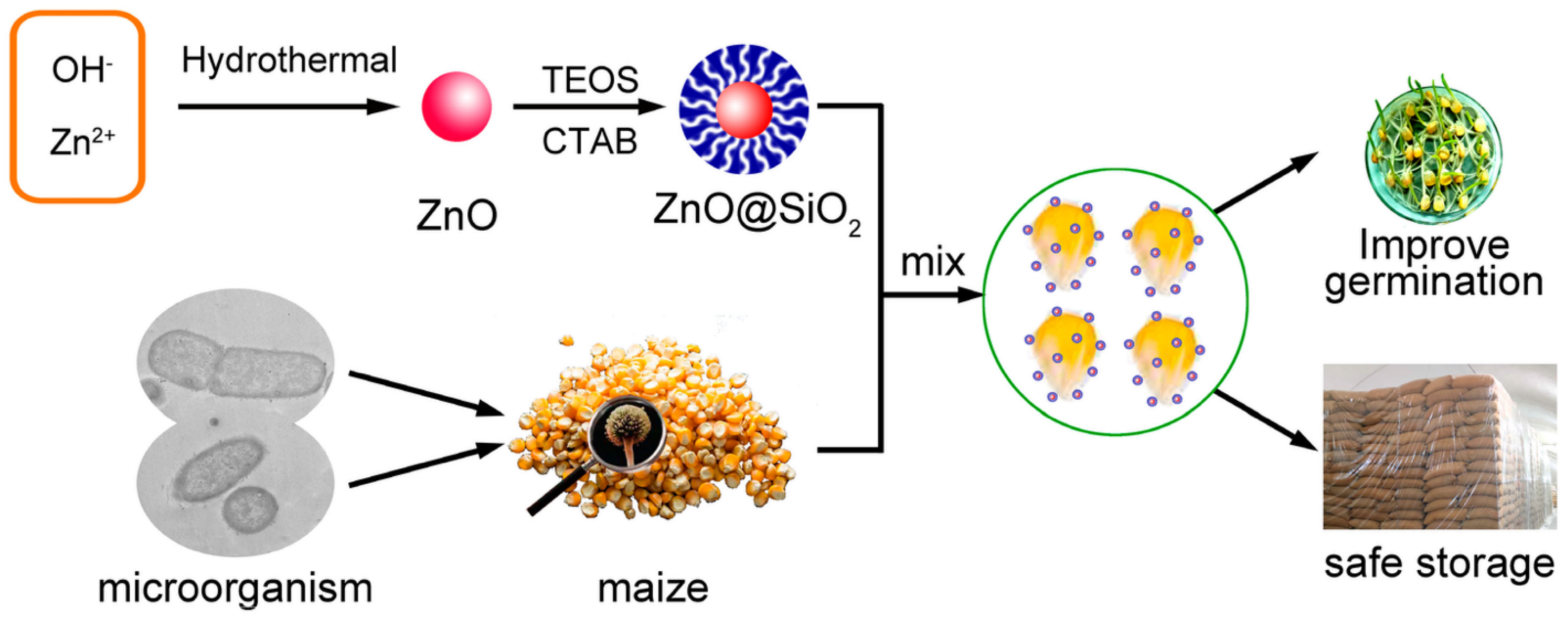
Application of ZnO@SiO₂ nanocomposites in maize protection and seed storage. Source: Zhou et al. (2023), reproduced under CC BY 4.0 license.

Zinc oxide nanoparticles (ZnO NPs), another promising class of nanomaterials, have been extensively researched for their antifungal capabilities. Their efficacy can be attributed to their unique physical and chemical properties, which allow them to generate reactive oxygen species (ROS) that damage fungal cells (Sun et al., 2018; Cruz-Luna et al., 2021). ZnO NPs have been shown to interfere with enzyme activities associated with cellular metabolism, resulting in inhibited growth and spore formation of pathogenic fungi (Sun et al., 2018; Pachaiappan et al., 2021). The contact of fungal cells with these nanoparticles typically leads to membrane damage, thus crippling their functional integrity and viability (Cruz-Luna et al.,2021). Moreover, novel synthesis techniques such as those involving plant extracts have been explored, which enhance the biocompatibility of ZnO NPs and reduce environmental impact, further appealing to eco-aware agricultural practices (Adebayo et al., 2021; Cruz-Luna et al., 2023). For instance, synthesized through hydrothermal methods involving zinc ions (Zn^+^) and hydroxide (OH^−^), followed by encapsulation with silica (TEOS) and a surfactant (CTAB), the ZnO@SiO_2_ nanostructures are designed to inhibit microbial contamination in stored maize (Zhou et al., 2023). Upon mixing with maize seeds, these nanoparticles protect against fungal and bacterial pathogens, improving seed viability and extending storage life (**Figure 3**; Zhou et al., 2023).

**Figure 4:**
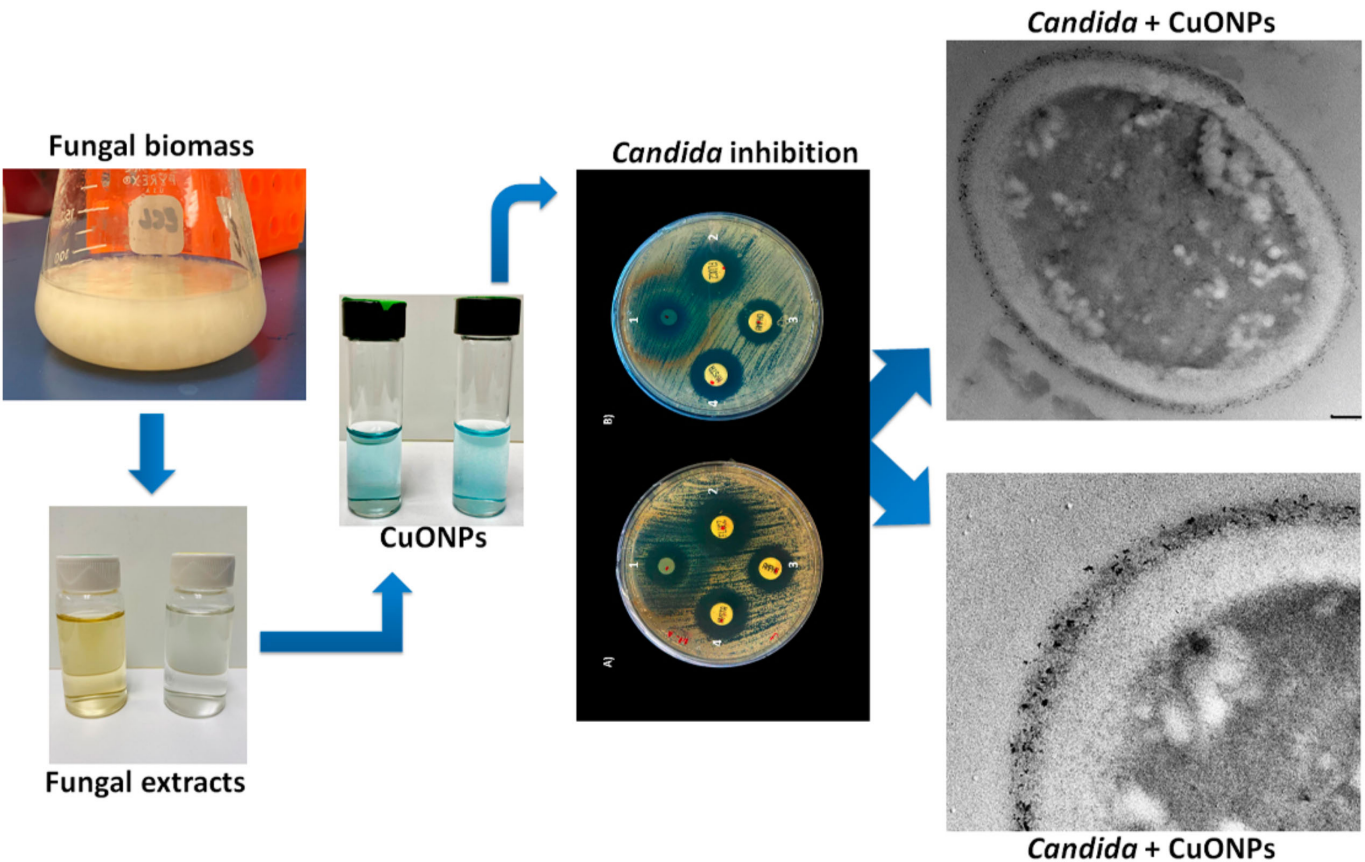
Fungal biosynthesis of copper nanoparticles (CuONPs) and their antifungal activity against Candida spp. Source: Garcia-Marin et al. (2022), reproduced under CC BY 4.0 license.

Equally significant are copper oxide nanoparticles (CuO NPs), which have demonstrated efficient antifungal effects, particularly against resistant strains (Zhao et al., 2020; Wahab et al., 2023). Their action revolves around generating oxidative stress within the target cells, which ultimately induces apoptosis (Zhao et al., 2020; Wahab et al., 2023). Numerous studies have corroborated the use of CuO NPs in agricultural settings, where they provide an alternative to synthetic fungicides, minimizing the environmental footprint and enhancing sustainable practices in fungal management (Sun et al., 2018; Pachaiappan et al., 2021). The potential utilization of these metallic nanoparticles in agroecosystems signifies a considerable shift toward more sustainable pest management strategies. **Figure 4** presents the green synthesis of copper oxide nanoparticles (CuONPs) using fungal biomass and extracts, followed by their application in inhibiting fungal pathogens, specifically *Candida* species (Garcia-Marin et al., 2022). The process begins with the extraction of bioactive compounds from fungal cultures, which are then used to synthesize CuONPs, as indicated by the color change in solution. The resulting nanoparticles exhibit strong antifungal effects, as demonstrated by the inhibition zones on agar plates and the ultrastructural damage to *Candida* cells observed under electron microscopy (Garcia-Marin et al., 2022; **Figure 4**). This eco-friendly nanobiotechnology approach holds promise for sustainable antifungal strategies in agriculture and clinical settings, offering a safer alternative to conventional chemical fungicides.

**Figure 5:**
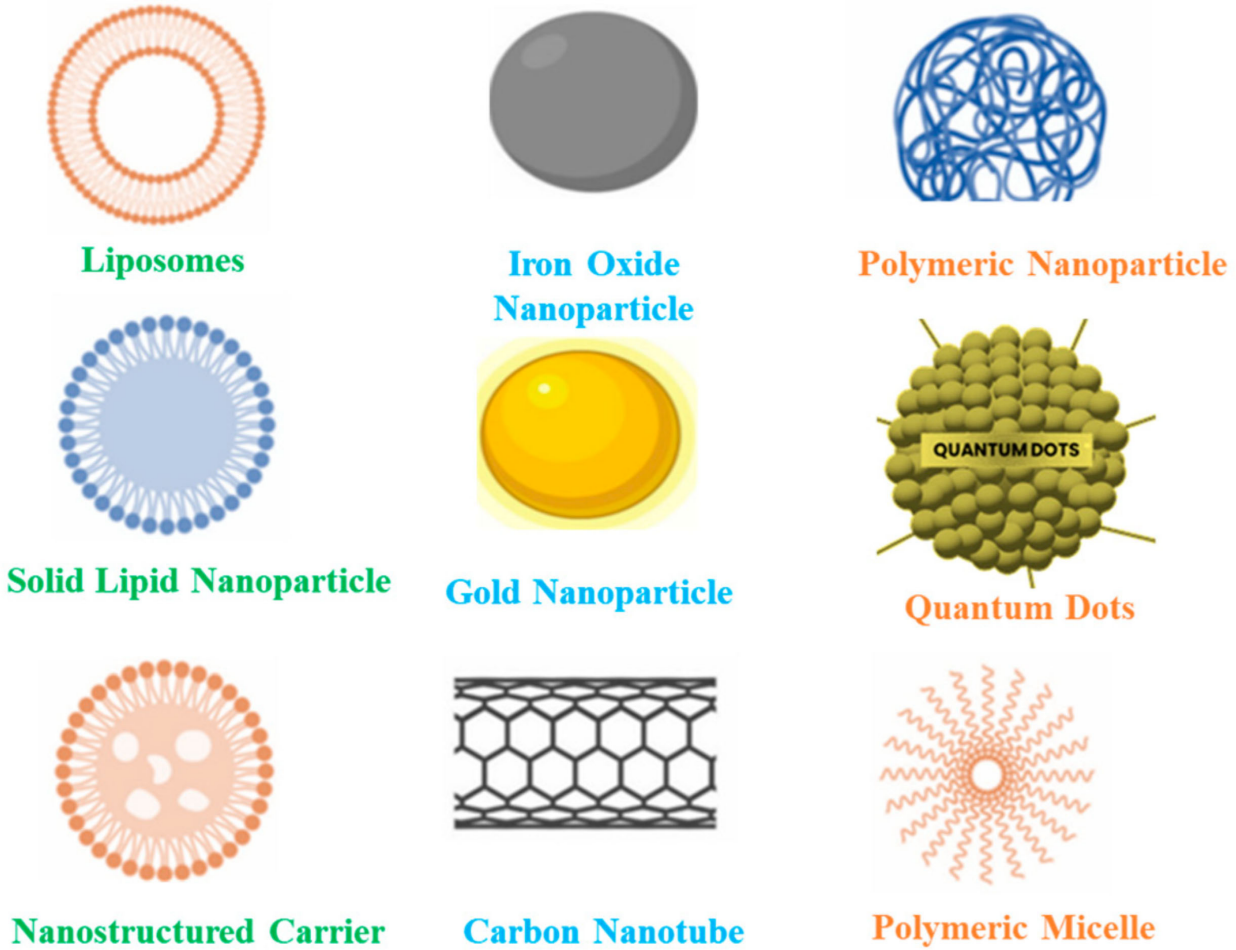
Some types and examples of nanocarriers. Sources: adapted from Hani et al. (2024), reproduced under CC BY 4.0 license.

Nanocarriers and controlled release systems have also emerged as innovative approaches to enhancing the delivery of antifungal agents. Some examples of nanocarriers include liposomes, solid lipid nanoparticles, gold and iron oxide nanoparticles, polymeric micelles, and carbon nanotubes (**Figure 5**). Nanocarriers are engineered nanoscale materials designed for the targeted delivery of active compounds, including agrochemicals, pharmaceuticals, and nutrients. Their high surface-area-to-volume ratio, tunable physicochemical properties, and ability to carry functional payloads make them highly versatile (Hani et al., 2024). By encapsulating antifungal agents within nanostructured carriers, the stability and bioavailability of these compounds can be enhanced, potentially improving their therapeutic efficacy and reducing systemic toxicity. While the concept of targeted delivery to fungal cells is promising, current scientific evidence supporting precise, pathogen-specific targeting remains limited and largely exploratory (Adebayo et al., 2021; Cruz-Luna et al., 2023). However, polymer-based nanocarriers have shown potential for controlled and sustained release of antifungal agents, helping to maintain localized drug concentrations over extended periods (Madkour, 2017; Alghuthaymi et al., 2015). This sustained release may reduce dosing frequency and side effects associated with conventional treatments, thereby improving therapeutic outcomes and patient compliance.

**Figure 6:**
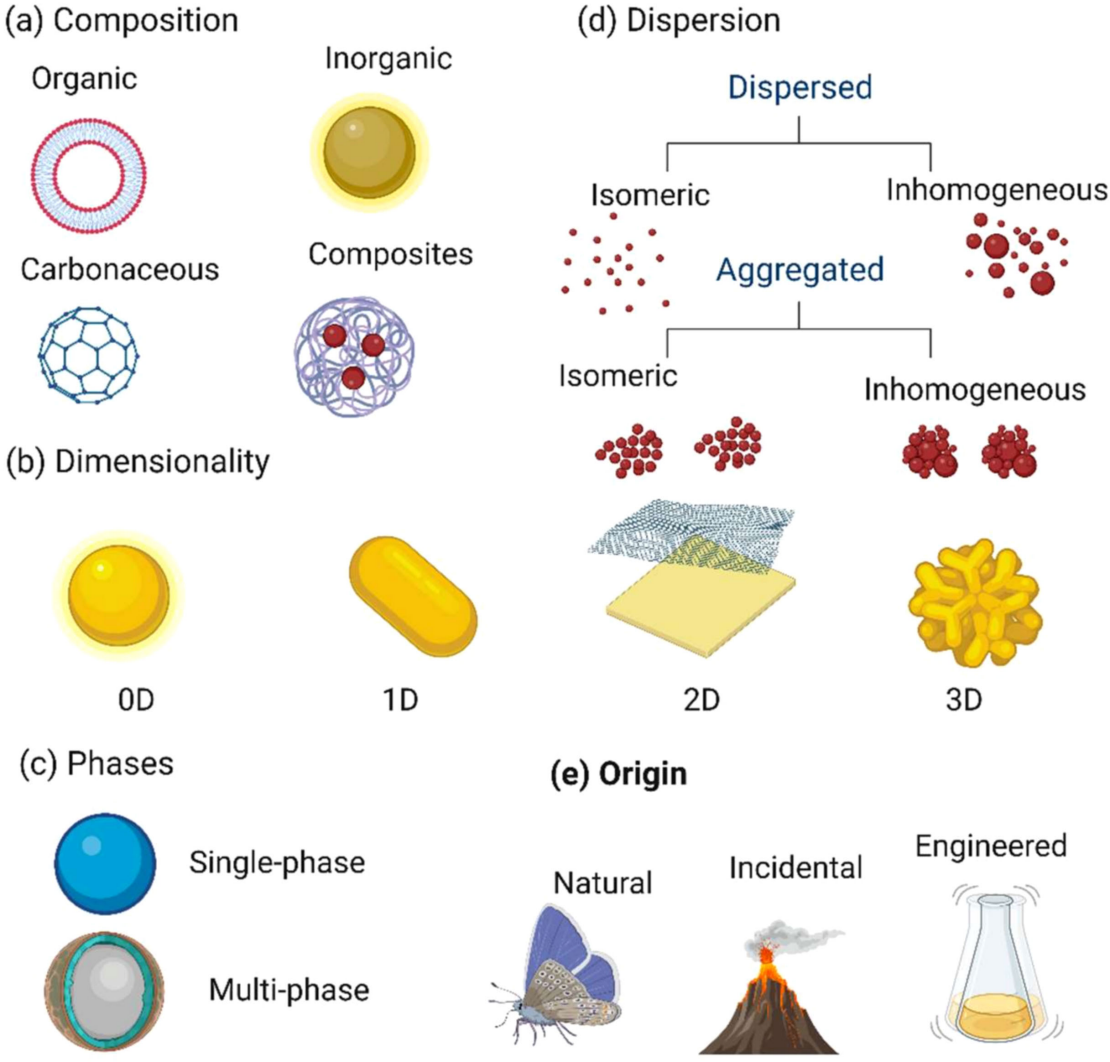
Comprehensive classification of nanoparticles. Source: Harish et al. (2022), reproduced under CC BY 4.0 license.

Nanostructured materials, including nanotubes and nanowires, provide additional avenues for inhibiting fungal pathogens. **Figure 6** presents the multi-dimensional framework for classifying nanostructured materials based on five key criteria according to Harish et al. (2022):

- Composition: Nanoparticles may be organic, inorganic, carbonaceous (e.g., fullerenes), or composites combining multiple materials.
- Dimensionality: Classification by shape and size includes zero-dimensional (0D, spherical), one-dimensional (1D, rod-like), two-dimensional (2D, sheet-like), and three-dimensional (3D, complex shapes).
- Phases: Nanoparticles can exhibit a single-phase structure or a multiphase architecture with core-shell or layered configurations.
- Dispersion State: Nanoparticles are categorized as dispersed or aggregated, with further distinction between isomeric and inhomogeneous forms in each case.
- Origin: Their source can be natural (e.g., biological processes), incidental (e.g., volcanic emissions), or engineered (e.g., laboratory synthesis).

The structures of these materials can interact with fungal cell surfaces, leading to the obstruction of essential metabolic pathways or even physical disruption through structural nanomechanical interactions (Dhillon et al., 2011; Rai et al., 2021). The unique aspects of these materials facilitate enhanced interaction at the cellular level, thereby improving their antifungal efficacy compared to traditional bulk materials (Cruz-Luna et al., 2023). Furthermore, the versatile nature of nanostructured materials enables their modification and functionalization with various antifungal agents, thereby expanding their application scope in agricultural biotechnology (Adebayo et al., 2021).

The mechanisms of action of these nanoscale materials also reveal intricate pathways through which fungal pathogens can be managed. The disruption of cell membranes by nanoparticles is a fundamental action that can be explained through the formation of reactive oxygen species and the interaction between nanoparticles and various cellular targets, including proteins and nucleic acids (Pachaiappan et al., 2021; Adebayo et al., 2021). Studies have illustrated how AgNPs induce morphological changes in fungal cells, contributing to their lethal effects and showcasing their potential as viable alternatives to synthetic fungicides in agriculture (Panchangam and Upputuri, 2019; Rai et al., 2021). The advantages of nanotechnology over traditional fungal control methods extend beyond enhanced efficacy. The use of nanoparticles can significantly mitigate the environmental and health risks typically associated with conventional fungicides. Research indicates that lower amounts are necessary to achieve the desired antifungal effects compared to traditional chemical agents, which can decrease chemical runoff and residual toxicity in ecosystems, thereby reinforcing ecological health (Wahab et al., 2023; Mohsen et al., 2022).

Additionally, their sustained-release properties can minimize the frequency of application and enhance treatment efficiency, even though precise, pathogen-specific targeting remains an aspirational goal in agricultural nanotechnology. Current formulations aim to prolong antifungal activity and improve stability rather than achieve selective action against individual pathogens (Cruz-Luna et al., 2021; Alghuthaymi et al., 2015). Moreover, as nanotechnology integrates into agricultural practices, compliance with increasingly stringent environmental regulations is facilitated, making the approach beneficial for crop protection and aligned with global sustainability goals (Sun et al., 2018; Adebayo et al., 2021). The application of nanotechnology in managing fungal pathogens is multifaceted, involving various types of nanoparticles, nanocarriers, and nanostructured materials that enhance efficacy and address the limitations of traditional methods. The ongoing research into these applications suggests a promising transformation in how we approach fungal infections in agriculture and potentially in clinical settings, heralding a future where nanotechnology could significantly enhance our ability to combat microbial threats.

## 3 Public health impacts of nanotechnology in agriculture

Integrating nanotechnology into agriculture is increasingly recognized as a transformative approach to address various public health challenges, notably through its role in reducing reliance on harmful pesticides, enhancing food safety, mitigating antibiotic resistance, and navigating associated environmental risks. The versatility of nanomaterials enables precise and controlled applications that minimize unintended human and ecological harm while enhancing disease control efficacy. However, alongside these benefits, potential risks associated with nanoparticle persistence, bioaccumulation, and cross-sectoral interactions must be carefully assessed. **Table 2** outlines the key public health domains influenced by nanotechnology in agriculture, detailing the roles and impacts of nano-enabled innovations, associated risks, and recommended strategies for mitigating these risks. This framework supports a balanced, evidence-based understanding of how nanotechnology can be harnessed to promote safer, more resilient, and health-conscious food systems.

**Table 2:** Public health impacts of nanotechnology in agriculture.

Public health area	Nanotechnology’s role	Impacts/Benefits	Potential risks	Mitigation strategies	References
Pesticide Reduction	Nanopesticides, controlled-release systems	Reduced human exposure to toxic chemicals; less environmental contamination	Accumulation of nanomaterials in soil/water; unknown long-term effects	Regulatory oversight, dose optimization, and ecotoxicological studies	Chaud et al. (2021)
Food Safety	Nano-enabled antifungal coatings and packaging	Prevention of mycotoxin contamination; extended shelf-life of produce	Ingestion of residual nanoparticles through food	Development of safe packaging materials, residue monitoring	Evivie et al. (2020); Oladeji et al. (2024)
Antimicrobial Resistance (AMR)	Alternative to antibiotics in crop protection	Reduced antibiotic use in agriculture; slower spread of AMR genes	Possible resistance development to nanoparticles	Rotation of nanoparticle types, integrated pest management (IPM)	Salam et al. (2023)
Zoonotic Disease Prevention	Improved crop health and food hygiene	Reduced fungal reservoirs that could transfer to animals/humans	Unknown cross-species interactions with nanomaterials	One Health–oriented research, cross-sector risk assessment	Ogwu and Izah (2023)
Occupational Health	Safer formulations and delivery methods	Reduced inhalation/dermal exposure for farmers and workers	Exposure to airborne nanoparticles during application	Use of personal protective equipment (PPE), safety training	Ogwu and Izah (2025)
Environmental Health	Lower pesticide runoff and targeted applications	Protection of water bodies, pollinators, and non-target organisms	Persistence of nanoparticles in ecosystems	Lifecycle assessment, biodegradable nanomaterials	Ogwu (2025)

### 3.1 Reducing the use of harmful pesticides

Nanotechnology offers innovative solutions that can significantly reduce dependency on traditional chemical pesticides, which are often associated with adverse environmental and health effects. This transition arises from the ability of nanomaterials to enhance the efficacy of pesticides by allowing for targeted delivery and controlled release, overcoming limitations of conventional formulations that often lead to over-application and soil contamination (Kim et al., 2017). For instance, nanopesticides are designed to maximize the effective concentration of active ingredients while minimizing dispersal in the surrounding ecosystem, reducing the chemical load in agricultural practices (Parveen et al., 2023; Izah and Ogwu, 2023). Human health benefits arise from decreased pesticide exposure, which is correlated with fewer pesticide-related illnesses among both agricultural workers and consumers. Reducing chemical residues in crops directly contributes to improved food safety standards, as consumers are more likely to purchase and consume products not laden with toxic residues. Studies indicate that lower chemical usage can diminish the risk of chronic health issues associated with pesticide exposure, including endocrine disruption and neurotoxicity (Kim et al., 2017). Thus, the technological shift towards nanopesticides is pivotal for environmental sustainability and advancing public health concerns related to contaminated food supplies.

### 3.2 Food safety and nanotechnology

The promise of nanotechnology extends into food safety, particularly through its applications in preventing fungal contamination in crops. Mycotoxins, produced by certain fungi, pose significant health risks as they can enter the food supply chain, leading to various health issues, including carcinogenic effects (Zhang et al., 2020; Nurtjahja et al., 2022). Nanotechnology offers mechanisms to mitigate this issue, such as the development of nanosensors that detect and monitor fungal pathogens in real-time, enabling timely intervention (Rahim et al., 2021). In addition to detection, nanomaterials can offer antifungal properties that inhibit the growth of pathogenic fungi, thereby safeguarding food quality (Parveen et al., 2023; Zhang et al., 2020). These advancements have shown promise in field applications and post-harvest treatment, enhancing the shelf life of agricultural produce by targeting mycotoxin-producing strains, such as *Aspergillus flavus* (Nurtjahja et al., 2022). The subsequent reduction in mycotoxin levels is correlated with improved public health outcomes. It mitigates foodborne illnesses and reduces associated healthcare costs, underscoring the importance of integrating nanotechnology into agricultural practices for enhanced food safety.

### 3.3 Mitigating antibiotic resistance in agriculture

Antibiotic resistance has escalated into a global public health crisis, extensively linked to agricultural practices that employ antibiotics for disease prevention and growth promotion (Economou and Gousia, 2015; Checcucci et al., 2020). The transference of antibiotic-resistant bacteria and genes through soil, water, and food systems poses severe health risks to human populations, necessitating alternative strategies to mitigate this threat (Ogwu and Izah, 2025). Current research highlights the potential of nanotechnology as a non-antibiotic solution aimed at controlling pathogens without exacerbating antibiotic resistance (Chang et al., 2014). Nanomaterials exhibit antimicrobial properties that can effectively reduce plant pathogenic loads, thus potentially replacing antibiotic treatments (Kischkel et al., 2020). Nanotechnology could minimize the selection pressures that lead to the emergence of resistant strains in both agricultural and human contexts. For instance, studies demonstrate that applying such nanomaterials can suppress bacterial growth in crops, thereby reducing the need for chemical inputs that contribute to the resistance problem (Rahim et al., 2021). Collaborative efforts between agricultural research and public health can ensure that advancements in nanotechnology are effectively harnessed to combat antibiotic resistance and promote a safer food system for consumers.

Unlike conventional fungicides, which often act through single, specific biochemical pathways, making them prone to resistance development, nanomaterials exert their antifungal effects through multiple simultaneous mechanisms (Rai et al., 2009; Lemire et al., 2013). These include physical interactions, such as membrane disruption, the generation of reactive oxygen species, and interference with intracellular components, including DNA and enzymes (Khan and Rizvi, 2014; Lemire et al., 2013). This mode of action makes it more difficult for fungal pathogens to adapt through mutation or selection of resistant strains. Additionally, the nanoscale size of these materials enables closer and more prolonged interaction with target cells, enhancing their fungicidal potency while reducing the likelihood of metabolic or efflux-based resistance. However, ongoing monitoring is essential, as overuse or improper application of nanomaterials could eventually select for tolerance mechanisms, especially under field conditions.

### 3.4 Environmental and human health risks of nanomaterials

As with any emerging technology, the deployment of nanotechnology in agriculture raises concerns regarding environmental and human health risks. The potential for exposure to nanomaterials through food, water, and soil necessitates rigorous assessment protocols to understand their biological interactions and possible toxicological effects (Yang et al., 2019; Kristanti et al., 2021; Izah and Ogwu, 2025). Current literature suggests that nanomaterials can enhance agricultural productivity and sustainability; however, their release into ecosystems poses unintended risks, encompassing both ecological and health dimensions (Ikhajiagbe et al., 2020; Zahid et al., 2022). Establishing safety protocols and regulatory frameworks that govern the use of nanomaterials in agriculture is imperative to mitigate these risks. Continuous research is essential to evaluate the long-term impacts of nanomaterial exposure on human health and the environment, ensuring that public health is prioritized in the integration of these technologies (Kim et al., 2017; Alazaiza et al., 2021). Collaboration among scientists, regulatory agencies, and policymakers will play a critical role in establishing robust frameworks that explore benefits while addressing potential hazards associated with nanotechnology in agriculture.

The intersection of nanotechnology and agriculture presents transformative opportunities for advancing public health through innovative, science-driven practices. By reducing reliance on conventional chemical pesticides, nanotechnology minimizes harmful environmental exposures and enhances food safety and quality. Moreover, the targeted and efficient delivery systems offered by nanoscale materials hold great promise in addressing the growing global challenge of antibiotic resistance by providing alternative mechanisms for disease control. These advancements also offer tools to mitigate post-harvest losses and extend food shelf life, further contributing to nutritional security. However, deploying nanotechnologies in agricultural systems must be cautiously approached. Comprehensive risk assessments, transparent regulatory frameworks, and ongoing interdisciplinary research are crucial to mitigating unintended ecological and health consequences. Ultimately, responsible innovation in agricultural nanotechnology can be a powerful lever for achieving more resilient, equitable, and sustainable food systems while protecting human and environmental health.

## 4 Nanotechnology in fungal disease prevention and control

Nanotechnology is increasingly recognized as a pivotal tool in revolutionizing fungal disease management across agricultural systems, through the early detection of fungal pathogens, the prevention of cross-contamination in food systems, and its role within integrated pest management (IPM) frameworks. These innovations provide enhanced precision in monitoring and controlling fungal threats, aligning with broader public health protection, environmental sustainability, and economic resilience goals. By enabling rapid diagnostics, reducing the reliance on chemical pesticides, and enhancing crop resistance, nanotechnology establishes itself as a transformative force in developing next-generation agricultural disease prevention strategies.

### 4.1 Early detection of fungal pathogens using nanotechnology

Nanotechnology represents a groundbreaking advancement in agricultural practices, particularly concerning the early detection of fungal pathogens that jeopardize crop yield and food safety. The development of nanosensors allows for the rapid identification of fungal pathogens, facilitating timely intervention and management of potential outbreaks (Ray et al., 2023; Parveen et al., 2023). Traditional disease detection methods often involve lengthy processes that delay necessary treatments, whereas nanosensors provide a swift and efficient alternative, enabling farmers to act promptly to mitigate losses (Singh, 2023; Jali et al., 2024). For instance, nanoscale biosensors can detect the presence of pathogens in crops through colorimetric or electrical conductivity changes, significantly reducing the conventional constraints associated with pathogen diagnostics (Nainnwal, 2025). The impact of early detection systems extends beyond immediate crop health; they contribute significantly to preventing the spread of diseases through meticulous monitoring of environmental conditions conducive to pathogen proliferation. Continuous monitoring of soil health and climate variables is made possible by incorporating nanotechnology into precision agricultural techniques. This continuous vigilance not only aids in the early identification of threat pathogens but also fosters improved crop resilience by enabling farmers to implement targeted pest and disease management strategies (Singh, 2023; Rani et al., 2024). Consequently, integrating these technologies enhances food security and ensures that agricultural outputs remain safe for consumption. The economic implications of employing such innovative detection systems are critical. Crop losses attributed to fungal infections can range between 20% and 40%, severely impacting agricultural sustainability and economic viability (Khan et al., 2021; Ajaz et al., 2024). By promoting rapid detection methodologies facilitated by nanotechnology, agricultural stakeholders can substantially enhance crop yields while reducing their dependency on harmful chemical treatments often used for disease control (Sundararajan et al., 2023; Ajaz et al., 2024). This shift to environmentally friendly practices fosters a more sustainable agricultural landscape and addresses growing public health concerns regarding chemical residues in food products.

### 4.2 Prevention of cross-contamination in food systems

In addition to early detection, nanotechnology plays a significant role in preventing cross-contamination in food systems throughout storage and transportation processes. The application of nanomaterials in food safety demonstrates promising capabilities in controlling the transmission of fungal diseases, which can severely compromise food quality and safety from farm to table (Scortichini, 2022; Jagadeeshkumar, 2025). Nanoparticles can be utilized in packaging materials to enhance their antimicrobial properties, thereby reducing the risk of pathogen growth during storage (Srivastava et al., 2024; Singh et al., 2025). For example, incorporating nanosilver into packaging materials has exhibited effective and practical activities, thereby mitigating the risks of cross-contamination during food transit (Mohanty et al., 2024; Mohammad et al., 2022).

Moreover, effective management of food storage practices, augmented by nanotechnology, ensures that fungi and other pathogens are kept at bay. By optimizing packaging technologies and integrating nanoscale solutions, the shelf life of perishable products can be extended, reducing food waste and enhancing public health. Soni et al., 2024). This supports the principles of sustainability and aligns with global goals related to food security amid increasing demand for safe food sources (Hasaneen, 2023; Rani et al., 2024). The adaptability of nanotechnology in the production and distribution phases of food systems makes it a valuable asset in addressing the dual challenges of ensuring quality and maintaining safety. The strategic application of nanotechnology in monitoring food safety encompasses the development of nanosensors, which provide real-time data on the condition of food products during transportation. These sensors can track temperature, humidity, and overall quality, swiftly addressing any deviations that could lead to contamination (Rani et al., 2024; Nainnwal, 2025). By employing this advanced technological framework, it becomes increasingly feasible to implement holistic food safety systems that encompass proactive contamination prevention from the farm to the consumer’s plate.

### 4.3 Integrated pest management and nanotechnology

Integrating nanotechnology into IPM practices offers transformative potential for sustainable pest and disease control measures in agriculture. Traditional pest management approaches often rely heavily on chemical pesticides, which have numerous adverse effects on the environment and human health. Scortichini, 2022; Parveen et al., 2023). However, nanotechnology presents a viable alternative by allowing for the development of nanoinsecticides and nanopesticides that are not only more effective but also require smaller quantities for pest control (Mehta et al., 2024; Bratovčić et al., 2023). This precision use mitigates the risks associated with chemical runoff and the development of resistant pest populations, thus promoting both environmental sustainability and agricultural productivity (Bratovčić et al., 2023; Sundararajan et al., 2023). Moreover, combining conventional IPM methods with nanotechnology enables a comprehensive approach to pest and disease management. Techniques such as crop rotation and biological control can be enhanced by nanoscale interventions that optimize pest monitoring and control. For example, nanosensors can detect pest populations and fungal pathogens, allowing farmers to deploy targeted biological control agents precisely when and where they are needed (Ajaz et al., 2024; Soni et al., 2024). This synergy between traditional practices and innovative technologies minimizes the overall pesticide load while maintaining effective control measures. Also, as crops face increasing biotic stresses from evolving pathogens, the role of nanotechnology in bolstering plant resilience cannot be overstated. Nanoparticles can enhance nutrient uptake and improve crop vitality, making plants less susceptible to pests and diseases (Jagadeeshkumar, 2025; Parveen et al., 2023). Implementing these technologies in conjunction with traditional agricultural practices creates a multi-faceted approach that acknowledges the complexities of agricultural ecosystems and promotes biodiversity while sustainably enhancing crop yield (Ray et al., 2023; Scortichini, 2022).

**Table 3** outlines key application areas where nano-enabled tools are deployed to enhance plant health and food safety. Each nanotechnological approach—from nanosensors to antimicrobial coatings and bio-based nanoformulations—serves distinct yet complementary functions. Collectively, they enable timely disease interventions, extend product shelf life, reduce post-harvest losses, and contribute to sustainable pest and disease management, including within organic farming systems. These innovations are not only improving the effectiveness and efficiency of disease control strategies but also enhancing the overall effectiveness of public health efforts. Still, they are also minimizing chemical inputs and aligning with global goals for safer, more sustainable agricultural practices.

**Table 3:** Applications of nanotechnology in fungal disease prevention and control in agriculture.

Application area	Nanotechnology tool/approach	Function	Benefits	Implementation example	References
Early Detection of Fungal Pathogens	Nanosensors, nano-biosensors, quantum dots	Detect specific fungal biomarkers or pathogens at early stages	Timely interventions, reduced disease spread, and improved crop yield	Use of graphene-based sensors for detecting Fusarium spp. in soil	Alam et al. (2024); Narware et al. (2025)
Contamination Prevention in Food Systems	Nano-coatings, nano-packaging, antimicrobial films	Prevent fungal growth during storage and transport	Enhanced food shelf-life, reduction in post-harvest losses, safer food products	Silver nanoparticle-infused films to inhibit mold in packaged grains	Rezghi Rami et al. (2024)
Prevention of Farm-to-Table Cross-Contamination	Nanomaterial-based surface sanitizers and coatings	Disrupt fungal spores on surfaces, packaging, and tools	Breaks the pathogen transmission chain, improves hygiene in the supply chain	TiO_2_ nanocoatings on harvest equipment surfaces	Rizou et al. (2020); Nazarov et al. (2023)
Integrated Pest and Disease Management (IPM)	Nanopesticides combined with biological and cultural methods	Deliver antifungal agents in combination with traditional control strategies	Reduced chemical input, synergistic effect, and sustainability	Chitosan nanoparticles used with biocontrol fungi to suppress Botrytis cinerea	Poznanski et al. (2023)
Disease Suppression in Organic Systems	Bio-based nanoformulations (e.g., essential oil nanoemulsions)	Use of natural substances in nanoform to inhibit pathogens	Complies with organic standards, enhances the efficacy of natural products	Clove oil nanoemulsion for controlling powdery mildew in vegetables	Singh et al. (2021)

## 5 Sustainability and innovative fungal nanotechnology in agriculture

The incorporation of nanotechnology into sustainable agricultural practices offers a transformative approach to addressing the challenges faced by modern agriculture. As the global population continues to expand and environmental constraints intensify, it becomes increasingly imperative to explore innovative methods that enhance crop productivity while minimizing environmental impact. At the forefront of this paradigm shift is the integration of nanotechnology, which has been recognized as a critical avenue for achieving sustainable agricultural practices. Phytonanotechnology leverages nanoscale interventions to optimize resource efficiency, enabling farms to produce more with fewer inputs, such as pesticides and fertilizers, while reducing their ecological footprint (Jiang et al., 2021; Tang et al., 2023). Nanotechnology promotes sustainability in agriculture primarily by enhancing the efficiency of agrochemical inputs. For instance, nanofertilizers and nanopesticides have been developed to deliver nutrients and pest control agents more precisely and effectively than conventional methods. These advanced formulations mitigate drawbacks such as nutrient leaching and pest resistance that accompany the excessive use of chemical fertilizers and pesticides. Nanoscale innovations enable the controlled release of these substances, ensuring that crops receive precisely what they need, which leads to improved agricultural outputs while reducing harmful runoff into surrounding ecosystems (Kumar et al., 2023; Mohanty et al., 2024).

Another pivotal aspect of nanotechnology in fostering sustainability is its ability to minimize the environmental footprint associated with agricultural practices. Nanomaterials can significantly reduce chemical runoff and soil contamination, as these innovations enable targeted applications where they are most needed, thereby decreasing the overall volume of chemicals applied. By enhancing the efficiency of water and nutrient usage, nanotechnology can lower the risk of soil degradation and ecological imbalances (Tripathi et al., 2023; El-Ramady et al., 2023; Iavicoli et al., 2017). This shift from broad-spectrum applications to targeted delivery represents a substantial movement towards environmentally friendly practices that can help maintain biodiversity and soil health.

Moreover, the efficiency gained through nanotechnology enhances crop resilience, particularly against fungal diseases and environmental stresses. Research indicates that nanomaterials can help bolster plant defenses, thereby increasing their resistance to biotic stresses, such as pathogens, and reducing the need for external interventions, like chemical fungicides. Such advancements contribute to agricultural sustainability by decreasing reliance on synthetic chemicals while enhancing the natural resilience of crops (Tyagi et al., 2023; Wang et al., 2024). This is particularly relevant in organic farming, as nanotechnology can equip organic growers with tools to manage pests and diseases effectively without compromising the principles of natural farming practices (Shang et al., 2019; Tang et al., 2023). While the potential for sustainable advancements through nanotechnology is immense, it also brings challenges concerning long-term sustainability. Introducing nanoparticles into agricultural systems raises critical considerations regarding their environmental impact, particularly their accumulation in ecosystems. Therefore, developing guidelines for the sustainable production and disposal of nanomaterials is vital. Addressing these environmental concerns proactively will help mitigate risks associated with their long-term use, ensuring that the benefits of nanotechnology can be realized without detrimental effects on the environment (Mahakham et al., 2017; Iavicoli et al., 2017).

The holistic view of how nanotechnology can revolutionize agriculture aligns with global narratives concerning food security, environmental health, and socio-economic development. With a worldwide population projected to reach 9.7 billion, agriculture must adapt to ensure sufficient food production while avoiding overexploitation of natural resources and compromising ecosystem integrity. Nanotechnology has offered significant advancements in crop yield and quality, aligning with sustainable agricultural goals by fostering environmentally responsible and economically viable practices (Aliev et al., 2021; Kumar et al., 2023; Gelaye, 2025). The implications of these innovations extend beyond crop enhancement; they address critical global objectives such as poverty alleviation, improved nutritional security, and the resilience of agricultural livelihoods against climate change (Aliev et al., 2021; Gelaye, 2025). The evidence supports the feasibility and utility of incorporating nanotechnology into agriculture as a promising approach toward meeting future agricultural demands sustainably and responsibly (Jiang et al., 2021; Kumar et al., 2023; El-Ramady et al., 2023).

Innovations made possible by nanotechnology closely resemble ecological agricultural concepts and climate-resilient practices, as they improve resource efficiency, reduce environmental pollution, and decrease dependency on traditional agrochemicals. **Table 4** presents key sustainability focus areas where nanotechnology contributes to improved environmental outcomes and agricultural productivity. From innovative delivery systems that limit pesticide overuse to biodegradable nanomaterials that mitigate pollution, these technologies present compelling solutions to some of the most persistent challenges in modern agriculture. However, realizing their full potential requires addressing critical issues related to environmental safety, regulatory clarity, cost-effectiveness, and equitable access, particularly in regions dominated by smallholders.

**Table 4:** Sustainability benefits of innovative nanotechnology for managing fungal diseases in agriculture.

Sustainability focus area	Nanotechnology innovation	Contribution to sustainability	Environmental and agricultural benefits	Challenges	References
Reduction in Pesticide Use	Nano-enabled targeted delivery systems, nanocapsules	Minimizes the overuse of conventional fungicides	Lower chemical runoff, reduced health risks, improved biodiversity	Requires formulation optimization and scale-up	Zhang et al. (2024)
Resource Efficiency	Smart nanocarriers, nano-fertilizer–fungicide hybrids	Increases nutrient/fungicide uptake and reduces wastage	Improved crop yield with fewer inputs (water, chemicals)	Potential cost and accessibility barriers for smallholder farmers	Yadav et al. (2023)
Pollution Mitigation	Biodegradable or green-synthesized nanomaterials	Replaces persistent agrochemicals with eco-friendly alternatives	Decreased soil and water contamination, safer ecosystems	Environmental degradation pathways of some nanomaterials remain unknown	Silva et al. (2021)
Improved Crop Resilience	Nano-priming and nano-coatings for seed and plant immunity	Enhances plant tolerance to fungal stress	Reduced need for external chemical interventions	Varies by crop type and ecological context	Kundu et al. (2024)
Support for Organic Farming	Nanoemulsions and bio-based nanoparticles (e.g., chitosan)	Aligns with organic principles while improving fungal control	Expanded options for organic growers, reduced post-harvest losses	Need for certification and policy clarity on nano-use in organics	Ruffo Roberto et al. (2019); Poznanski et al. (2023)
Long-Term Ecosystem Health	Controlled-release nanoformulations	Prevents the accumulation of excess chemicals in the environment	Promotes soil microbial health and long-term productivity	Long-term nanoparticle fate and interactions need more research	Iavicoli et al. (2017)

## 6 Challenges and limitations of fungal nanotechnology in agriculture

The exciting potential of fungal nanotechnology in agriculture is matched by various challenges that must be addressed for successful implementation. The application of nanotechnology in fungal disease management faces a range of technical, regulatory, environmental, and social challenges that must be systematically addressed to ensure responsible and equitable deployment. **Table 5** categorizes key limitations across these domains, highlighting issues such as high production costs, regulatory gaps, environmental persistence, and public skepticism. These barriers impact the scalability and sustainability of nano-enabled solutions, raising significant concerns about long-term safety, ecological balance, and access equity. One of the primary technical and practical challenges facing fungal nanotechnology is the scalability and cost-effectiveness of nanomaterial production. Effective deployment in agriculture requires efficient synthesis methods that can produce high-quality nanoparticles in large quantities without prohibitive costs. As highlighted by Kumar et al. (2023), the practical application of nanotechnology in agriculture relies on innovation and the development of scalable production processes that strike a balance between quality and cost efficiency.

**Table 5:** Challenges and limitations of nanotechnology applications for fungal disease management in agriculture.

Challenge category	Specific limitation	Explanation	Implications	Possible solutions	References
Technical Challenges	High production cost	Synthesis of high-purity, stable nanoparticles is often expensive and energy-intensive	Limits affordability and adoption by smallholder farmers	Promote green synthesis and low-cost fabrication techniques	Singh et al. (2023); Sundararajan et al. (2023)
	Environmental instability	Nanomaterials may degrade or lose efficacy under field conditions (e.g., UV light, rain, pH)	Reduced field performance and inconsistent disease control	Develop formulations with improved stability and protective coatings	Chaud et al. (2021); Martínez et al. (2020)
	Delivery and targeting issues	Difficulty in achieving uniform application and precise targeting of pathogens	May lead to reduced efficacy or waste of materials	Use of innovative delivery systems and nanocarriers	Patra et al. (2018); El-Tanani et al. (2025)
Regulatory and Safety Concerns	Lack of standardized regulations	Absence of clear national or international guidelines for nano-agriculture	Regulatory uncertainty hinders approval and public trust	Develop coordinated policies through interdisciplinary collaboration	Kosoe et al. (2023); Kumar and Ogwu (2025)
	Limited toxicological data	Insufficient understanding of long-term effects on humans, soil, and ecosystems	Raises safety concerns for users and consumers	Conduct comprehensive risk assessments and life cycle analyses	Sharma et al. (2022); Ogwu et al. (2025c)
Environmental Impact	Persistence in ecosystems	Some nanoparticles may accumulate in soil or water and resist degradation	Disruption of microbial communities and soil health	Focus on biodegradable and eco-friendly nanomaterials	Rai et al. (2021)
	Effects on non-target organisms	Nanoparticles may unintentionally harm pollinators, beneficial microbes, or aquatic species	Biodiversity loss and ecological imbalance	Perform targeted studies on non-target impacts and refine application methods	Yamini et al. (2023)
Social and Ethical Issues	Public perception and awareness	Concerns about “nano” technologies in food production and the lack of farmer training	Hesitancy to adopt and possible market resistance	Increase public education, transparency, and stakeholder engagement	Bostrom and Löfstedt (2010)
	Access and equity	Technological and economic disparities between high-income and low-income regions	Risk of widening global agricultural inequality	Support equitable access and international collaboration in nano-agriculture research	Iavicoli et al. (2017)

Furthermore, the physical and chemical stability of these nanoparticles under various environmental conditions poses a significant challenge. Factors such as UV radiation, temperature extremes, and humidity can affect the efficacy of nanomaterials in agricultural applications, creating the need for effective stabilization techniques (Tang et al., 2023). Mohanty et al. (2024) noted that without durability under such conditions, the anticipated benefits of using nanomaterials to enhance agricultural productivity could be compromised.

Consequently, the development of regulatory frameworks surrounding fungal nanotechnology is crucial, as existing regulations often fail to address the unique aspects of nanomaterials adequately. The complexity and novel properties of nanoparticles necessitate the establishment of targeted policies that incorporate comprehensive risk assessments explicitly tailored to these materials (Abd–Elsalam, 2024). Currently, regulatory gaps hinder the effective deployment of nanotechnology in agriculture, particularly in terms of safety and environmental impact assessments. There is a pressing need for global standards that can facilitate the safe application of these advanced technologies, which, as outlined by Abd–Elsalam (2024), is essential for ensuring public health while maintaining agricultural productivity and sustainability. Regulatory bodies lack a cohesive strategy to manage the potential risks associated with introducing nanoparticles into farming practices, creating an environment of uncertainty among stakeholders (Prasad et al., 2017).

Furthermore, research endeavors must prioritize understanding the fate and transport of nanomaterials in agricultural systems, as highlighted by Vijayakumar et al. (2022), to effectively evaluate their potential environmental impact. An interdisciplinary approach, combining fields such as toxicology, ecology, and nanotechnology, is crucial for addressing these complex challenges directly and developing strategies to mitigate associated risks (Elmeanawy et al., 2022). This is especially critical given that emerging technologies like fungal nanotechnology must ensure ecological integrity and sustainability while enhancing productivity. Failure to build public confidence may stall the integration of innovative technologies into agricultural practices, despite their potential to improve productivity and sustainability. Technical challenges associated with production and stability, regulatory gaps, safety and environmental concerns, and public perception issues collectively form a complex barrier that must be addressed through coordinated efforts from policymakers, researchers, and practitioners. This multifaceted approach is crucial to ensuring that the promise of fungal nanotechnology can be fully realized in promoting sustainable agricultural practices.

## 7.0 Stakeholder engagement, equity, and adoption pathways for nanotechnology in crop protection

### 7.1 Why equity and engagement matter in nanotechnology adoption for crop protection

Nanotechnology presents a transformative opportunity in agricultural science, offering innovative solutions to some of the pressing global challenges that farmers face, particularly those in low- and middle-income countries (LMICs). The efficacy of such scientific innovations hinges on their technological advancements and their ability to engage with and be inclusive of the diverse stakeholder landscape within agricultural systems. Smallholder farmers, disproportionately affected by adversities such as fungal crop diseases, represent a critical demographic that must be explicitly considered in the broader nanotechnology adoption dialogue. Despite their pivotal role in global food security, these farmers often lack direct access to the advancements that nanotechnology affords, primarily due to socio-economic barriers and entrenched inequalities (Shang et al., 2019; Khundi et al., 2025). Integrating nanotechnology into crop protection can enhance sustainability, resilience, and productivity in agriculture (Ogwu and Kosoe, 2025). This claim is supported by research indicating that nanomaterials can improve pest control and increase crop yields by efficiently delivering nutrients and pesticides (Liu et al., 2021; Singh and Kumar, 2024; Mohanty et al., 2024). However, while these technologies may offer promising results on a macro scale, they tend to perpetuate existing inequities if the most vulnerable populations, such as smallholder farmers, are not actively engaged in discussions surrounding their development and implementation (Mohanty et al., 2024; Gelaye, 2025). Moreover, the knowledge gap in the application of these technologies can exacerbate inequitable access, leading to a situation where only those with resources can capitalize on the advancements of nanotechnology, leaving marginalized groups further behind (He et al., 2019).

### 7.2 Barriers to adoption

Several barriers hinder the adoption of nanotechnology in agriculture, particularly among smallholder farmers in LMICs. One of the most formidable obstacles is the high cost of developing and deploying nanomaterials (Kansotia et al., 2024; ElSayed et al., 2025). While nanotechnology can potentially reduce input costs over time, the initial investments for smallholder farmers can be prohibitive, particularly when the market is still predominantly driven by larger agricultural enterprises (Khundi et al., 2025; Rathore et al., 2024). However, it is worth noting that consumers often express hesitation towards novel technologies perceived as risky, underscoring a need for educational initiatives that responsibly communicate nanotechnology’s benefits and risks (Obahiagbon and Ogwu 2023; 2024). This hesitance is often rooted in limited public understanding, uncertainty about long-term health or environmental impacts, and broader distrust of technological interventions in food systems. Without proactive outreach and transparent communication, these concerns may hinder the adoption of promising nanotechnologies, particularly in regions where regulatory frameworks are still in development.

Hence, despite the potential cost savings associated with improved efficiency and reduced pesticide usage, the price tag attached to these materials can discourage uptake, posing an insurmountable barrier for resource-constrained farmers (Liu et al., 2021; Kumar et al., 2023). Additionally, the limited availability of extension services and trained professionals in rural areas exacerbates the challenge of integrating nanotechnology in agriculture. Many smallholder farmers rely heavily on local extension services for information and education regarding new agricultural practices. However, these services are often inadequate or absent in many regions, resulting in a significant knowledge gap regarding nanotechnology and its potential benefits (Singh et al., 2024; Lallawmkimi et al., 2025). This has resulted in a pervasive distrust of novel technologies, as farmers are often uncertain about the implications and safety of incorporating such materials into their farming practices (Mohanty et al., 2024; Gelaye, 2025).

Moreover, regulatory frameworks around using and disseminating nanotechnology in agricultural products can be slow to develop, delaying the introduction of innovative solutions and creating uncertainty among farmers (ElSayed et al., 2025; Rathore et al., 2024). Many countries lack legislation or guidance on registering nano-enabled agricultural products, which hampers local adoption initiatives. In addition, issues related to gender inequity in technology access further complicate the adoption landscape, as women—who constitute a significant portion of the agricultural workforce in many LMICs—often face additional barriers to accessing training and resources (Akinhanmi et al., 2023).

### 7.3 Stakeholder involvement and participatory approaches

Engaging diverse stakeholders in the research and development process is crucial for overcoming barriers to the adoption of nanotechnology in crop protection. Key stakeholders include farmers, cooperatives, extension officers, NGOs, and policymakers, all bringing unique perspectives and expertise (Singh et al., 2024). Initial stages of any technological development must prioritize participatory approaches that involve farmers directly, especially in context-sensitive regions where local knowledge and practices are invaluable. Strategies such as participatory technology design, where farmers actively contribute to creating and adapting new technologies, have shown promise in ensuring that innovations cater to actual needs rather than imposed solutions (Singh and Kumar, 2024; Tan et al., 2023). Farmer-led field trials are another effective strategy for fostering engagement and trust. Farmers can directly assess the advantages of nanotechnology applications by experimenting with them in controlled environments, which helps them make more informed decisions (Mohanty et al., 2024; Lallawmkimi et al., 2025). This farmer-centered approach builds capacity and encourages knowledge sharing within communities, enhancing overall trust in the technologies being introduced. Incorporating community feedback into product development processes is crucial for aligning these innovations with the practical realities farmers face. Solutions developed in close collaboration with end-users tend to have higher rates of acceptance and efficacy (Mohanty et al., 2024; Gelaye, 2025). Moreover, such participatory approaches promote transparency and accountability in the adoption of new technologies, fostering a sense of shared ownership among local communities, which can be instrumental in achieving equitable access to nanotechnology in agriculture (Elemike et al., 2019; Singh et al., 2024).

### 7.4 Equity in access to nanotechnology

Addressing equity in access to nanotechnology necessitates concerted efforts to bridge the digital divide and ensure effective technology transfer among marginalized communities. One promising avenue to foster inclusive access is the establishment of public-private partnerships to create subsidized programs that support smallholder farmers in adapting to nanotechnology (Campos et al., 2023; Singh and Kumar, 2024). These partnerships can leverage resources and expertise from both sectors, enhancing capacity-building efforts to empower farmers with the knowledge and tools necessary to implement these innovations. Open-access platforms that disseminate knowledge and resources related to nanotechnology in agriculture can further democratize this knowledge. These platforms can contribute to a broader understanding of the uses and advantages of nanotechnology by providing farmers and extension agents with readily available information, training materials, and research findings (Mohanty et al., 2024; Singh et al., 2024). Additionally, local manufacturing of nanomaterials can help reduce costs and increase accessibility while promoting sustainable practices by minimizing the environmental footprint associated with transporting these materials across borders (Kumar et al., 2023; Akinhanmi et al., 2023).

Moreover, targeted policies focusing on gender equity in access to technological advancements are essential for ensuring that all farmers, regardless of gender, can benefit from nanotechnology. Interventions such as specialized training programs, mentorships, and financial resources tailored to women farmers can empower them to harness the benefits of these innovations and contribute to a more equitable agricultural landscape (Gowda et al., 2024). The potential of nanotechnology can be fully realized by addressing these complex issues through inclusive practices and equitable regulations, ensuring that no one is left behind in the agricultural revolution (Kubiak et al., 2022; Rathore et al., 2024).

## 8 Future directions and policy recommendations for adopting nanotechnology solutions for managing fungal diseases in crops

As nanotechnology continues to revolutionize the management of plant fungal pathogens, its long-term success hinges on a strategic and inclusive roadmap that bridges scientific innovation with practical application. The future of fungal disease control will require advanced nanomaterials and delivery systems, supportive regulatory environments, robust public engagement, and cross-sector collaboration.

### 8.1 Advances in nanotechnology research for fungal disease control

The prospective integration of nanotechnology in combating fungal pathogens reveals significant potential, particularly in the agricultural sector, where traditional methods are proving insufficient. Novel nanomaterials, such as gold nanoparticles (AuNPs), have demonstrated promising antifungal properties by inhibiting key biological processes in pathogens like *Candida albicans* (Yu et al., 2016). Ongoing investigations into the mechanisms of action of these nanomaterials are crucial; for instance, AuNPs inhibit the activity of H^+^-ATPase, a crucial enzyme for fungal pathogenicity, demonstrating efficacy in preventing biofilm formation and host cell invasion (Yu et al., 2016). Further research is exploring other nanomaterial types, such as antimony-based compounds, which exhibit antifungal activity against resistant strains of pathogens like *Cryptococcus neoformans*, underscoring a broader trend toward using metal-based nanoparticles to combat multidrug-resistant fungal infections (Gerasimchuk et al., 2022).

Future applications will likely focus on sustainably harnessing these technologies for agricultural purposes. Adopting eco-friendly nanomaterials could reduce reliance on chemical fungicides, which negatively impact environmental health and contribute to the development of resistance in fungal populations (Zaki et al., 2021). For example, the use of zinc oxide nanoparticles synthesized through biotechnological means has demonstrated efficacy in controlling soil-borne pathogens, offering a green approach to disease management in crops such as cotton (Zaki et al., 2021). As these technologies evolve, they could significantly enhance crop resilience and yield while mitigating health risks associated with conventional antifungal treatments. Collaborative efforts among researchers, agricultural stakeholders, and policymakers would be essential to facilitate the incorporation of such innovations into standard farming practices. Public health protection will become increasingly intertwined with advancements in nanotechnology. The ongoing innovations in nanoparticle therapies, particularly in combating infections caused by *Candida auris*, highlight a critical need for optimized therapeutic options in both clinical and agricultural settings (Izadi et al., 2024). These technologies promise to revolutionize infection control by providing effective targeted therapies against increasingly resistant fungal strains. Therefore, continuous investment in research and the translation of laboratory findings into real-world applications will be vital to harnessing the full potential of nanotechnology in the fight against fungal diseases.

### 8.2 Policy framework for nanotechnology in agriculture

The rapid advancements in nanotechnology necessitate the establishment of comprehensive regulatory frameworks to ensure the safe integration of nanomaterials into agricultural practices. The significant benefits that nanotechnology offers for enhancing plant disease control must be balanced with potential risks to human health and the environment (Fisher and Denning, 2023). Current regulatory guidelines fail to adequately address the complexities associated with nanotechnology, particularly concerning their potential to interact unpredictably with biological systems. Effective policy frameworks must emerge from collaborations among agricultural stakeholders, health authorities, and researchers to prioritize public health while promoting innovation. For a successful regulatory landscape, transparent protocols should be established to evaluate the safety and efficacy of nanomaterials in agriculture. Priority should be given to research on the environmental impacts, bioaccumulation, and toxicity of nano-enabled products before they are widely deployed (Zhu et al., 2023).

Additionally, ongoing surveillance and monitoring practices will be necessary to assess the effectiveness and safety of these new technologies over time, particularly as pathogens evolve and resistance mechanisms emerge. Thus, these combined efforts would create a dynamic and responsive regulatory environment that could adapt to the evolving landscape of agricultural nanotechnology. Furthermore, international cooperation is crucial for developing coherent policies that address the global nature of both agricultural trade and pathogens. Global standards could facilitate the movement and adoption of innovative nanomaterials across borders while maintaining consistency in safety measures and efficacy assessments. Harmonizing these regulations can promote trust among stakeholders and consumers alike, ensuring that the implementation of nanotechnology in agriculture is conducted responsibly.

### 8.3 Public awareness and education on nanotechnology

Public understanding of and education about nanotechnology’s role in plant disease control are essential for the successful adoption of these innovations. Informing farmers and agricultural workers about the advantages and safe practices related to nanotechnology can empower them to make informed choices in their agricultural practices. Community engagement initiatives, encompassing workshops and training sessions, could serve as platforms to disseminate knowledge and foster dialogue about the benefits and risks associated with nanotechnology (Fisher et al., 2022). Awareness campaigns should focus on demystifying nanotechnology and articulating its benefits in enhancing crop resilience and reducing dependency on traditional, potentially harmful fungicides. Farmers can be educated about specific applications, such as using biogenic nanoparticles to enhance plant disease resistance while minimizing environmental impacts. This will enhance farmers’ capacity to manage fungal diseases effectively and encourage sustainable practices that safeguard public health and environmental safety.

Moreover, the transparency of information is crucial in enhancing public trust. Stakeholders in the agricultural sector must engage in open discussions regarding the regulatory frameworks governing nanotechnology, ensuring that all stakeholders have a voice in decision-making processes. Collaborations with educators, governmental bodies, and scientists can establish a comprehensive information network that boosts public confidence in these emerging technologies. Extensive educational materials and resources should be readily available to various communities engaging with agricultural practices to foster informed decision-making. This would aid farmers in the immediate application and encourage young researchers to explore the potential of nanotechnology further. As public awareness grows, the collective acceptance and application of nanotechnology in sustainable agriculture are likely to flourish, leading to healthier crops, improved public health outcomes, and enhanced food security.

## 9 Conclusion

Nanotechnology holds transformative potential in managing plant fungal pathogens, marking a critical shift from traditional chemical-based approaches toward more precise, sustainable, and health-conscious solutions. This paper outlines that various nanomaterials—including metallic nanoparticles, nanocarriers, and green-synthesized formulations—exhibit potent antifungal activity through membrane disruption, oxidative stress induction, and targeted delivery. These innovations not only enhance crop protection and yield but also reduce the reliance on harmful pesticides, contributing directly to improved food safety, environmental quality, and public health outcomes. However, the promise of nanotechnology must be tempered with caution. Challenges such as scalability, regulatory gaps, environmental persistence, and the potential for unintended health impacts must be addressed through rigorous interdisciplinary research, transparent policymaking, and the development of global safety standards. Only through a balanced approach that embraces both innovation and responsibility can the full benefits of agricultural nanotechnology be realized.

In light of climate change, growing global food demands, and new disease concerns, nanotechnology is expected to have a significant impact on agriculture in the future. We preserve plant health and advance more public health objectives by incorporating nanotech solutions into eco-friendly disease prevention techniques, early disease detection platforms, and sustainable farming systems. Nanotechnology has the potential to significantly transform the way we produce, preserve, and consume food as scientific understanding and legal frameworks evolve, making the agricultural system safer, healthier, and more resilient for everyone.
